# MicroRNA-644a promotes apoptosis of hepatocellular carcinoma cells by downregulating the expression of heat shock factor 1

**DOI:** 10.1186/s12964-018-0244-z

**Published:** 2018-06-14

**Authors:** Wenjin Liang, Yong Liao, Zeming Li, Yan Wang, Siqi Zheng, Xiaochen Xu, Fulin Ran, Bo Tang, Zhenran Wang

**Affiliations:** Department of Gastrointestinal Surgery and Hepatobiliary Surgery, Guilin Medical University, Affiliated Hospital, Guilin, Guangxi 541001 People’s Republic of China

**Keywords:** Hepatocellular carcinoma, miR-644a, HSF1, BH3-only protein, Apoptosis

## Abstract

In this study, we investigated the role of microRNA-644a (miR-644a) in the growth and survival of hepatocellular carcinoma (HCC) cells. MiR-644a levels were lower in HCC tissues than in adjacent peri-cancerous tissues (*n* = 135). MiR-644a expression was inversely correlated with heat shock factor 1 (HSF1) expression, tumour diameter and TNM stage. Moreover, HepG2 and SMMC-7721 cell lines showed lower miR-644a expression than normal L-O2 hepatocytes. MiR-644a overexpression in HepG2 and SMMC-7721 cells increased apoptosis by downregulating HSF1. Dual luciferase reporter assays confirmed the presence of a miR-644a binding site in the 3’-untranslated region (3’-UTR) of HSF1. Xenograft tumours derived from SMMC-7721 cells transfected with a miR-664a mimic showed less growth than tumours derived from untransfected controls. Protein chip analysis revealed that miR-644a-overexpressing SMMC-7721 and HepG2 cells strongly expressed pro-apoptotic BH3-only proteins, such as BID, BAD, BIM, SMAC, Apaf-1 and cleaved caspases-3 and -9. These findings suggest miR-644a promotes apoptosis in HCC cells by inhibiting HSF1.

**Electronic supplementary material:**

The online version of this article (10.1186/s12964-018-0244-z) contains supplementary material, which is available to authorized users.

## Introduction

Hepatocellular carcinoma (HCC) is the primary malignancy of the liver and is the third leading cause of cancer deaths worldwide [1]. Despite significant progress in the diagnosis and treatment strategies, survival is extremely poor in HCC patients due to high recurrence rates [2, 3]. Therefore, there is an urgent need to discover new mechanisms that aid early diagnosis and treatment of HCC.

MicroRNAs (miRNAs) are a class of small endogenous non-coding RNAs that regulate gene expression by inhibiting mRNA transcription or translation [4, 5]. Moreover, miRNAs modulate growth, survival and other biological characteristics of tumour cells [6]. Several miRNAs have been identified as potential diagnostic and prognostic biomarkers for HCC; moreover, multiple miRNAs have shown potential as therapeutic targets in HCC [7]. Previously, miR-644a was identified as a tumour suppressor miRNA that inhibited oesophageal cancer cell proliferation [8]. It also inhibited breast cancer cell proliferation and drug resistance [9]. However, the role of miR-644a in HCC is unknown. In a preliminary screening using TargetScan and miRanda analysis, we found a putative miR-644a binding site in the 3'-UTR of HSF1 (Liang et al., unpublished observation). This finding suggests that HSF1 might be a potential direct target gene for miR644a.  Therefore, HSF1 was selected as the target gene for experimental analysis.

Heat shock factor 1 (HSF1) is a protective factor that is induced during various stress conditions including extrinsic environmental stress or intrinsic age-related deterioration [10]. HSF1 can also regulate protein homeostasis and degradation as well as malignant melanoma through the RAS-MEK signalling pathway [11]. Our research has shown that miR-644a expression is inversely correlated with HSF1 expression and promoted apoptosis in HCC cells by inhibiting HSF1, revealing a novel mechanism for miR-644a-mediated HCC progression that involves regulation by HSF1.

## Results

### Low miR-644a expression negatively correlates with HSF1 expression, tumour diameter and TNM stages in HCC

**Fig. 1:**
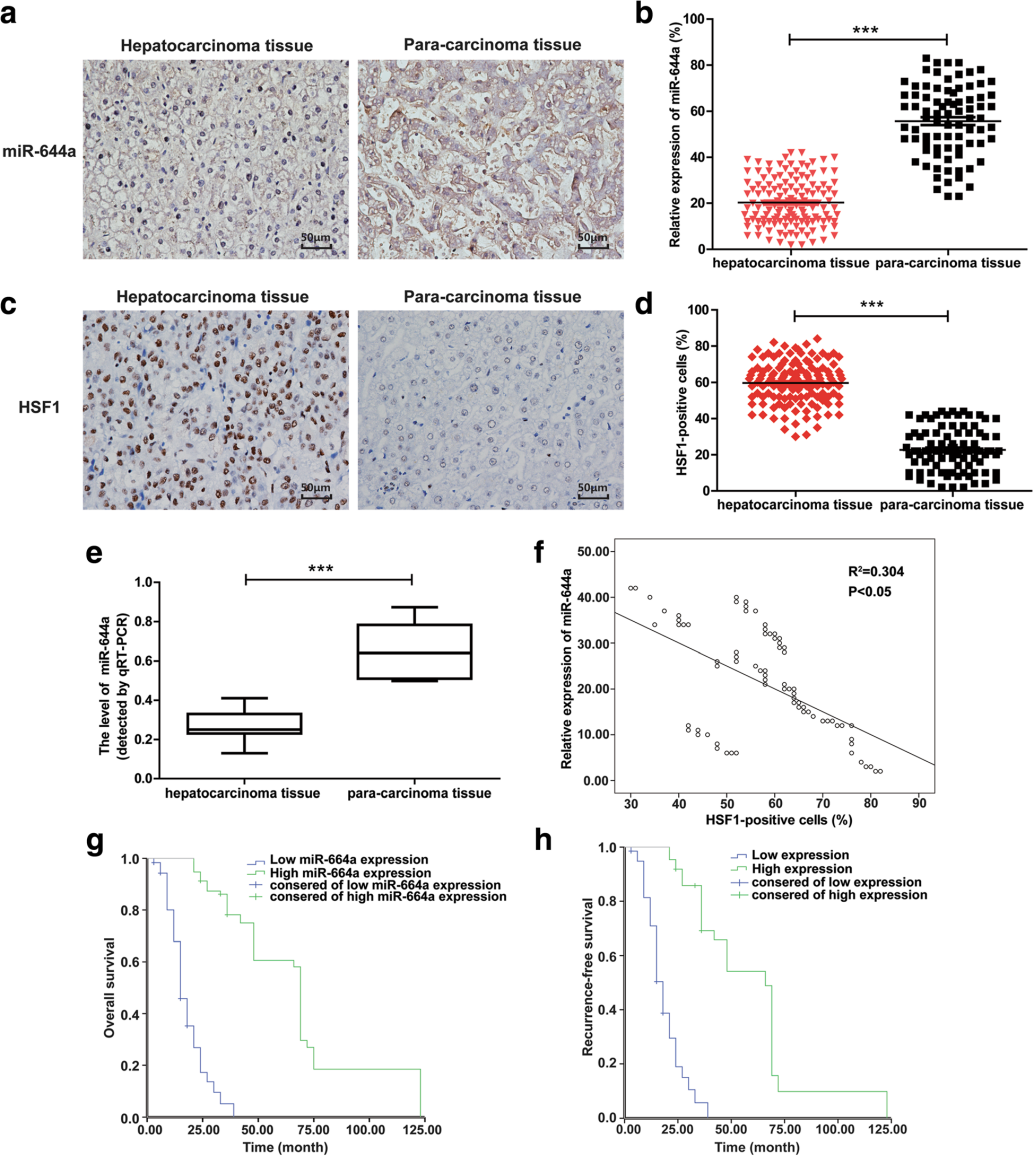
Low expression of miR-644a in HCC tissues negatively correlates with HSF1 expression and a poor prognosis. (a) Representative images (× 400) show in situ hybridization (ISH) of miR-644a in HCC and adjacent peri-cancerous liver tissues. (b) Quantitative analysis of miR-644a expression in HCC and adjacent peri-cancerous liver tissues (*n* = 135/group) based on ISH. Note: *** denotes *P* < 0.001 when compared to adjacent tissues. (c) Representative IHC images (× 400) show HSF1 expression in HCC and adjacent peri-cancerous tissues (n = 135/group). (d) Quantitative analysis of HSF1 expression in HCC and adjacent peri-cancerous liver tissues (n = 135/group) based on IHC. Note: *** denotes P < 0.001 when compared to adjacent tissues. (e) Quantitative RT-PCR analysis of miR-644a expression in HCC and adjacent peri-cancerous liver tissues (n = 135/group). Note: *** denotes P < 0.001 when compared to adjacent tissues. (f) Spearman’s test results show low miR-644a expression that negatively correlates with high HSF1 expression in HCC tissues (R^2^ = 0.304, *P* < 0.05). (g-h) Kaplan-Meier survival analysis demonstrates a correlation between miR-644a expression in HCC patients and their overall survival (g) and recurrence-free survival (h)

We performed in situ hybridization (ISH) and qRT-PCR analysis of miR-644a levels in surgically resected cancer and peri-cancerous liver tissues from 135 HCC patients. We observed lower expression of miR-644a in HCC tissues than in adjacent peri-cancerous liver tissues (*P* < 0.01, Fig. 1A–B). We also observed decreased miR-644a expression in HCC tissues (Fig. 1E).

Next, we analysed the correlation between miR-644a expression and the clinicopathological characteristics of the HCC patients. Based on the ROC curve analysis, we subdivided the 135 HCC patients into high- and low-miR-644a-expressing groups (area under the curve (AUC) = 0.837, *P* < 0.05; Additional file 1: Figure S1A), and did the same for HSF1 (AUC = 0.902, *P* < 0.05; Additional file 1: Figure S1B). Moreover, the expression of miR-644a was negatively correlated with HSF1 expression (*P* = 0.004), tumour diameter (*P* = 0.001) and TNM stage (*P* = 0.001; Table 1).

**Table 1:** Correlations berween miR-644a expression and clinicopathological parameters in 135 hepatocellular carcinoma patients

Variables	miR-644a expression		Total	*P* value
	Low (72)	High (63)		
Age (y)				
< 60	21(48%)	23(52%)	44	0.368
≥ 60	51(56%)	40(44%)	91	
Gender				
Male	48(59%)	34(41%)	82	0.134
Female	24(45%)	29(55%)	53	
Tumor diameter (cm)				
< 5	20(33%)	39(67%)	59	0.001^a^
≥ 5	52(68%)	24(32%)	76	
AFP (ng/ml)				
< 20	26	25	51	0.401
≥ 20	46	38	84	
Tumor differentiation				
Well	16(44%)	20(56%)	36	0.195
Moderate	27(53%)	15(36%)	42	
Poor	29(51%)	28(49%)	57	
TNM stage				
I-II	25(39%)	39(61%)	64	0.001^a^
III-IV	47(66%)	24(34%)	71	
HSF1 expression				
Low	13(34%)	25(66%)	38	0.004^a^
High	59(61%)	37(39%)	96	

*Abbreviation*: *HSF1* heat shock factor 1, *AFP* alpha-fetoprotein.

^a^Significant p value

Immunohistochemical analysis revealed higher HSF1 expression in HCC tissues than in adjacent peri-cancerous tissues (*P* < 0.01, Fig. 1C–D). Moreover, miR-644a expression was negatively correlated with HSF1 levels in HCC (Fig. 1F). Therefore, we postulated that miR-644a negatively regulates HSF1 expression.

### Prognostic significance of miR-644a expression in HCC patients

We further analysed the prognostic significance of miR-644a expression in HCC patients based on their 5-year survival rates. Kaplan-Meier survival curves showed that HCC patients with low miR-644a expression were associated with lower overall survival (14 months vs. 68 months; *P* < 0.001) and recurrence-free survival (18 months vs. 65 months; P < 0.001) than HCC patients with high miR-644a expression (Fig. 1G–H). Multivariate Cox regression analysis showed that low expression of miR-644a (relative risk = 0.350; *P* = 0.001) was an independent predictor of poor prognosis in HCC patients (Table 2). These results demonstrate that decreased expression of miR-644a correlates with poor prognosis in HCC patients.

**Table 2:** Multivariate Analysis with a Cox Proportional Hazards Regresssion Model

Variable	Univariate Analysis			Multivariate Analysis		
	RR	95% CI	P	RR	95% CI	P
Age < 45 years (VS. > 45 years)	1.023	0.422–2.480	0.960	1.300	0.479–3.523	0.608
Gender male (VS. female)	1.016	0.553–1.867	0.958	0.939	0.410–2.148	0.881
Tumor diameter < 2 cm (VS. > 2 cm)	0.726	0.384–1.373	0.324	0.886	0.361–2.177	0.792
AFP < 20 ng/ml (VS. > 20 ng/ml)	1.144	0.616–2.124	0.670	2.232	1.007–4.948	0.048
Tumor number Solitary (VS. Multiple)	0.699	0.364–1.343	0.283	0.407	0.153–1.082	0.072
Tumor differentiation Well or moderate (VS. poor)	1.378	0.716–2.650	0.337	0.834	0.340–2.051	0.693
TNM stage I-II (VS. III-IV)	0.568	0.287–1.124	0.104	0.567	0.229–1.404	0.220
miR-644a Low (VS. High)	0.379	0.183–0.784	0.009^a^	0.379	0.146–0.982	0.048^a^

*Abbreviation*: *RR* relative risk, *CI* confidence interval, *AFP* alpha-fetoprotein.

^a^Significant p value

### Correlation between miR-644a and HSF1 expression in HCC cell lines

**Fig. 2:**
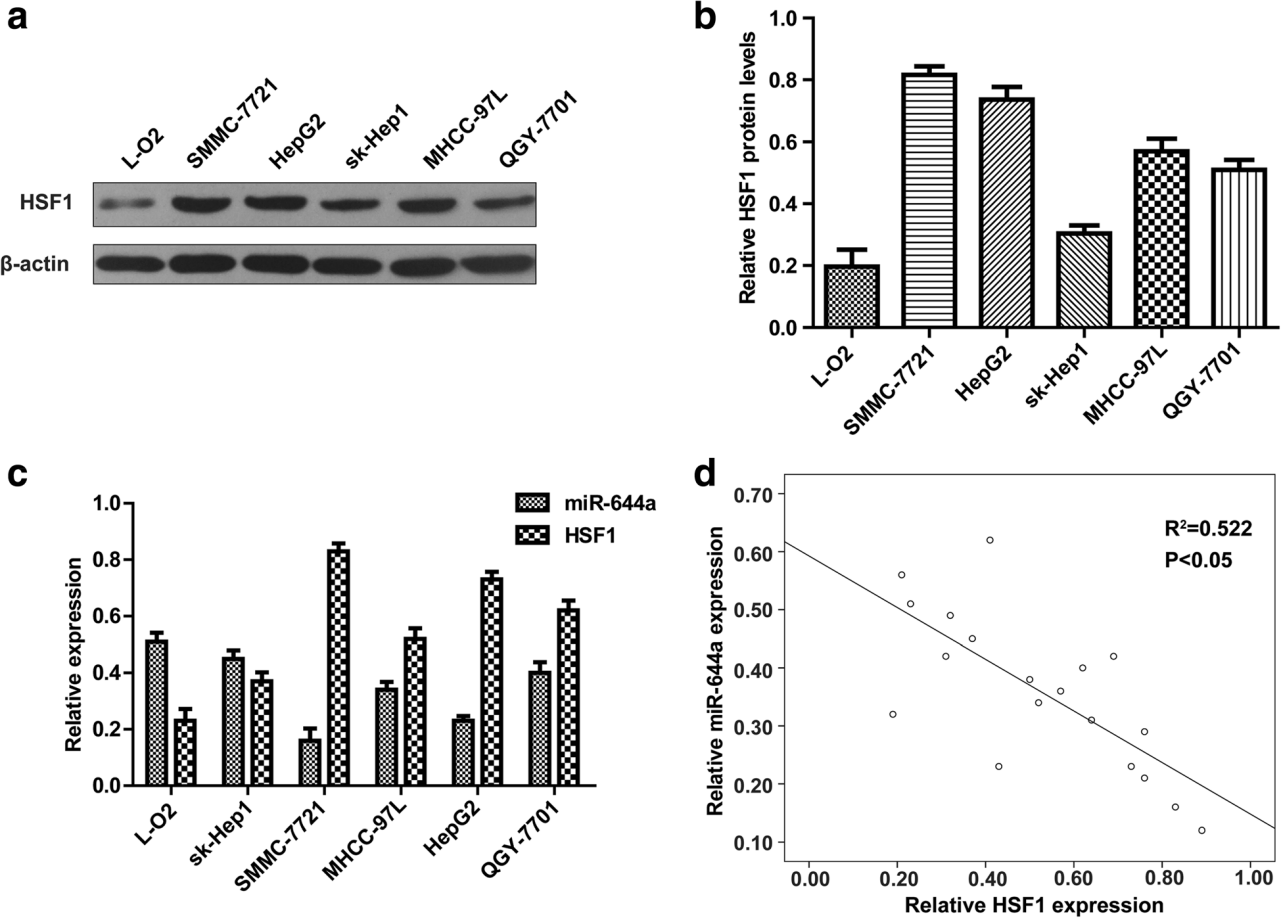
Correlation between miR-644a and HSF1 expression in HCC cells. (**a**) Quantitative RT-PCR analysis of miR-644a expression in HCC cell lines, HepG2, SMMC-7721, sk-Hep1, MHCC-97 L, QGY-7701 and the normal liver cells, L-O2. (**b**) Representative western blot shows HSF1 protein expression in the HCC cell lines HepG2, SMMC-7721, sk-Hep1, MHCC-97 L, and QGY-7701 and the normal liver cells, L-O2. (**c**) Quantitative RT-PCR analysis of HSF1 mRNA expression in the HCC cell lines, HepG2, SMMC-7721, sk-Hep1, MHCC-97 L, and QGY-7701 and the normal liver cells, L-O2. (**d**) Spearman’s analysis shows a correlation between miR-644a and HSF1 expression in the HCC cell lines (R^2^ = 0.522, *P* < 0.05). Note: Data are represented as the mean ± SD of three independent experiments and were analysed by a paired t-test

We verified the correlation between miR-644a and HSF1 by analysing their expression in multiple HCC cell lines and normal hepatocytes by qRT-PCR and western blotting, respectively. QRT-PCR analysis showed that miR-644a expression was lower in HepG2 and SMMC-7721 cells than in normal hepatocytes, L-O2, and the HCC cell lines sk-Hep1, MHCC-97 L and QGY-7701 (*P* < 0.05; Fig. 2A). Conversely, the mRNA and protein levels of HSF1 were higher in HepG2 and SMMC-7721 cells than in L-O2, sk-Hep1, MHCC-97 L and QGY-7701 cells (P < 0.05, Fig. 2B–C). Spearman’s correlation analysis showed that miR-644a levels were negatively correlated with HSF1 mRNA levels (*P* < 0.05, Fig. 2D).

### High miR-644a levels inhibit proliferation and promote apoptosis of HCC cells

**Fig. 3:**
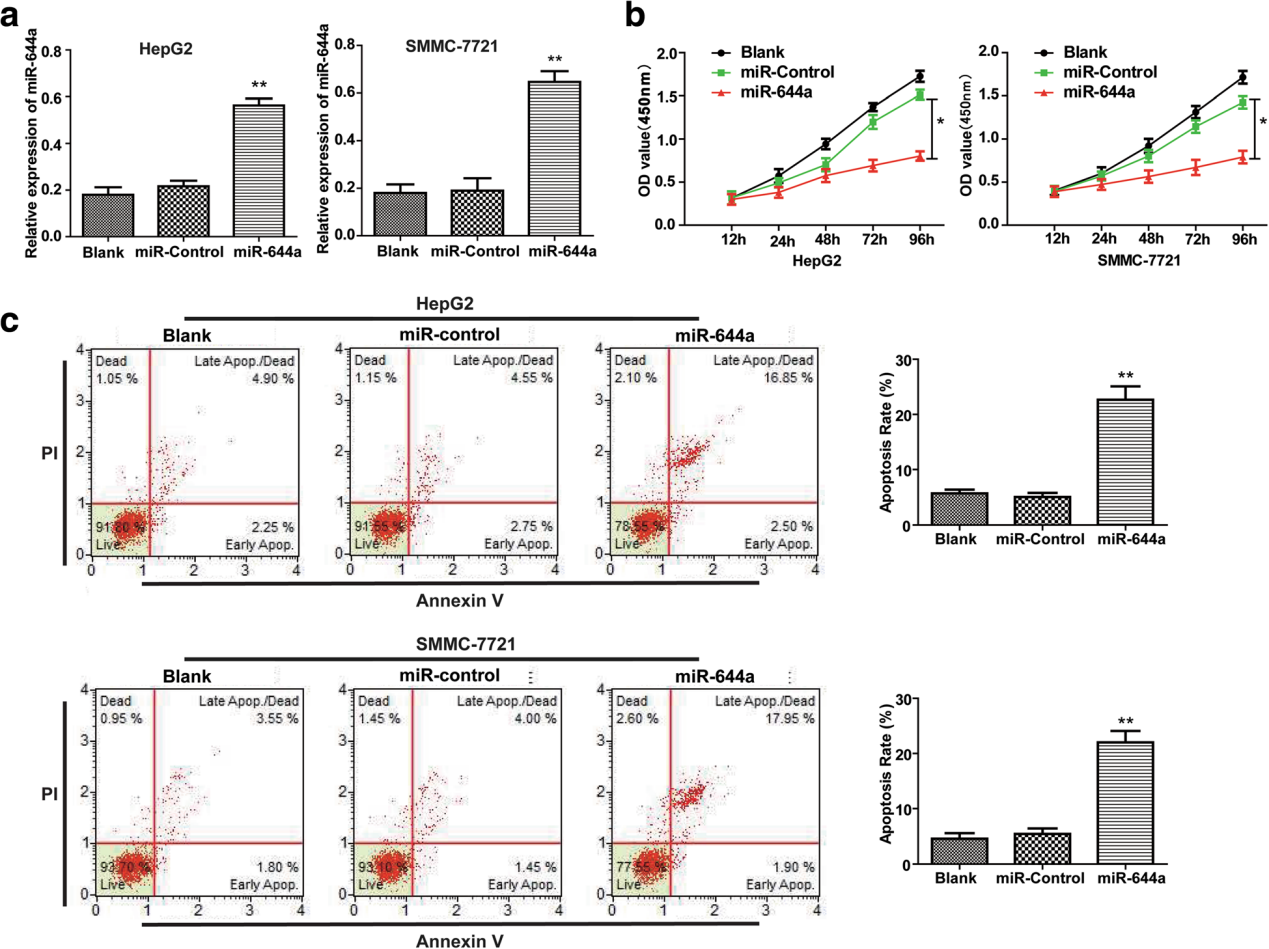
MiR-644a upregulation inhibits proliferation and promotes apoptosis of HCC cells. (**a**) Quantitative RT-PCR analysis of miR-644a expression in control and miR-644a mimic-transfected HepG2 and SMMC-7721 cells. (**b**) The CCK8 assay shows proliferation in the control and miR-644a mimic-transfected HepG2 and SMMC-7721 cells. (**c**) Flow cytometry analysis of percent apoptosis (AnnexinV^+^ PI^+^) in the control and miR-644a mimic-transfected HepG2 and SMMC-7721 cells. Note: * denotes P < 0.05 and ** denotes *P* < 0.01 compared to controls

Next, we investigated the role of miR-644a in proliferation and survival of HCC cells by transfecting miR-644a mimics into HepG2 and SMMC-7721 cells (Fig. 3A). The CCK-8 assay showed that miR-644a over-expression decreased the proliferation of HepG2 and SMMC-7721 cells more than in the corresponding controls (Fig. 3B). We further analysed the effects of miR-644a over-expression on apoptosis of HepG2 and SMMC-7721 cells by Annexin V/PI double staining. We observed that HepG2 and SMMC-7721 cells transfected with miR-644a mimics showed increased apoptosis compared with the corresponding controls (Fig. 3C). These results suggest that miR-644a negatively regulates proliferation and apoptosis of HCC cells.

### MiR-644a down-regulates HSF1 expression by binding to the HSF1 3’UTR

**Fig. 4:**
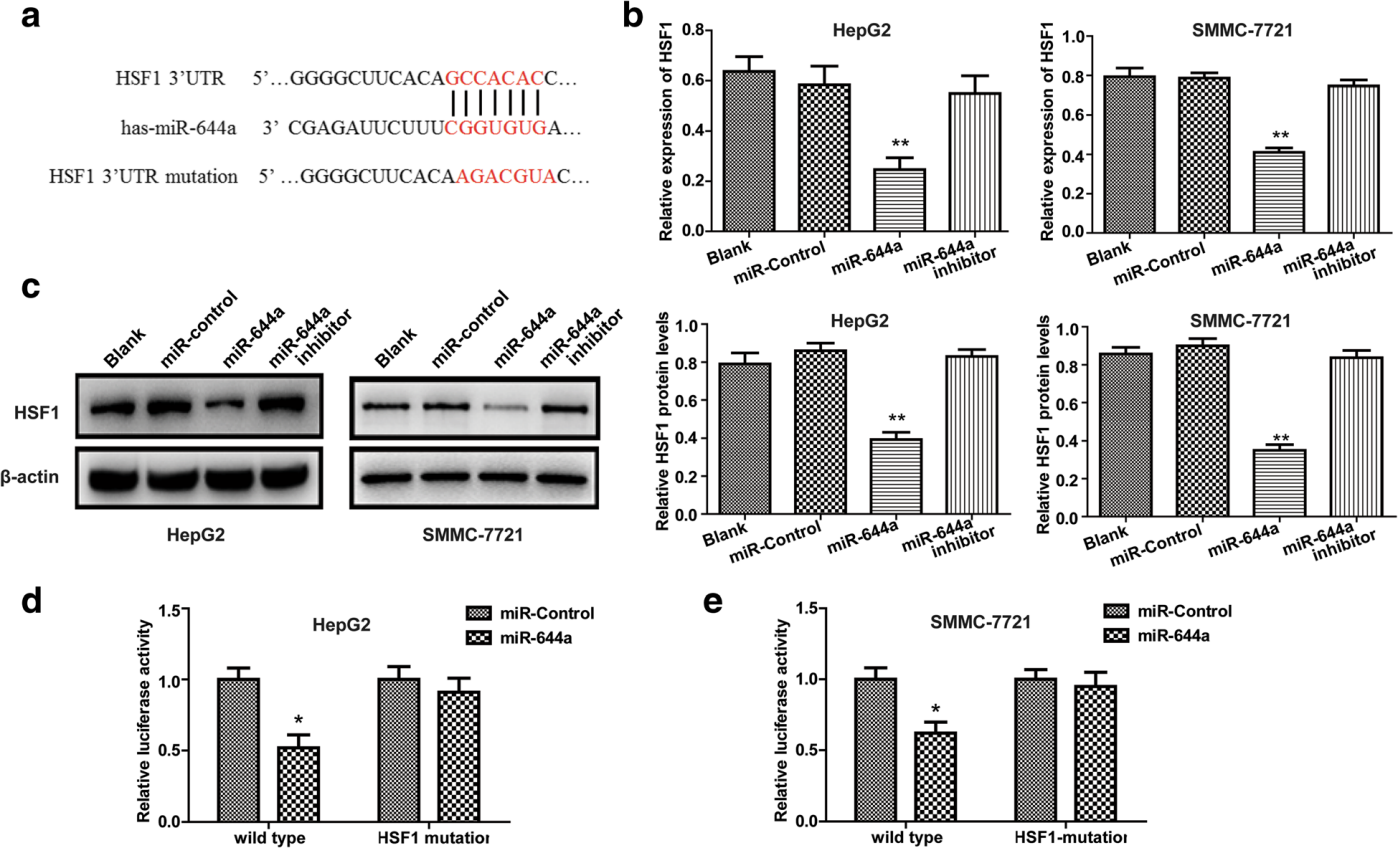
MiR-644a downregulates HSF1 expression by binding to its 3’UTR. (**a**) Targetscan and miRanda analysis show putative miR-644a binding sites in the 3’UTR of HSF1. (**b**) Quantitative RT-PCR analysis of HSF1 mRNA expression in HepG2 and SMMC-7721 cells co-transfected with miR-644a mimic and wild type or mutant HSF1. (**c**) Representative western blot shows HSF1 protein expression in HepG2 and SMMC-7721 cells co-transfected with miR-644a mimic and wild type or mutant HSF1. (**d**–**e**) Relative luciferase activity in HepG2 and SMMC-7721 cells co-transfected with miR-644a mimic and wild type or mutant HSF1. Note: * denotes *P* < 0.05 and ** denotes *P* < 0.01 compared to controls

TargetScan and miRanda analysis showed a putative miR-644a binding site in the 3’-UTR of HSF1 (Fig. 4A). Therefore, to prove that HSF1 was the target gene of miR-644a, we analysed HSF1 mRNA and protein levels in HepG2 and SMMC-7721 cells transfected with miR-644a mimics or miR-644a inhibitors. We observed that the miR-644a inhibitor increased HSF1 mRNA and protein expression, whereas the miR-644a mimic decreased HSF1 mRNA and protein levels in HepG2 and SMMC-7721 cells (Fig. 4B). Then, we investigated the correlation between miR-644a and HSF1 by using a dual luciferase reporter assay after co-transfecting HepG2 and SMMC-7721 cells with the luciferase reporter vector containing wild-type (wt) or mutant (mut) HSF1–3’UTR segments and miR-644a mimic or control miRNA. We observed a decreased relative luciferase activity in HCC cells co-transfected with miR-644a mimic and wild-type HSF1 3’UTR, whereas relative luciferase activity was normal in cells co-transfected with mutant HSF1 3’UTR and miR-644a mimics (Fig. 4D–E). These results showed that miR-644a down-regulates HSF1 expression.

### HSF1 promotes proliferation and survival of HCC cells

**Fig. 5:**
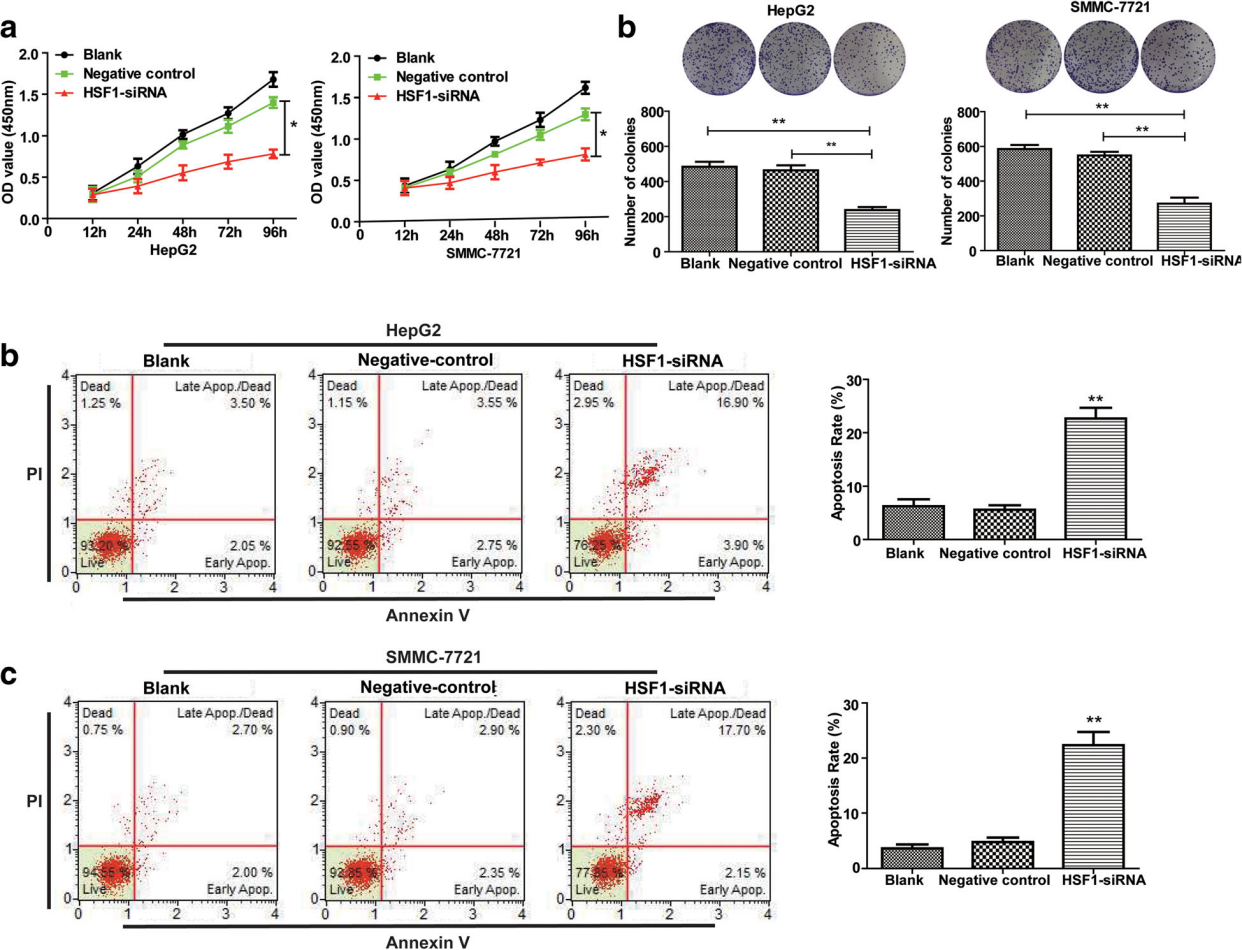
HSF1 silencing decreases proliferation and increases apoptosis of HCC cells. (**a**) CCK8 assay shows proliferation of HepG2 and SMMC-7721 cells transfected with control or HSF1-siRNA. (**b**) Colony formation assay shows the total number of colonies formed by HepG2 and SMMC-7721 cells transfected with control or HSF1-siRNA. (**c**) Flow cytometry analysis shows apoptosis of HepG2 and SMMC-7721 cells transfected with control or HSF1-siRNA. Note: * denotes *P* < 0.05 and ** denotes *P* < 0.01 compared to controls

**Fig. 6:**
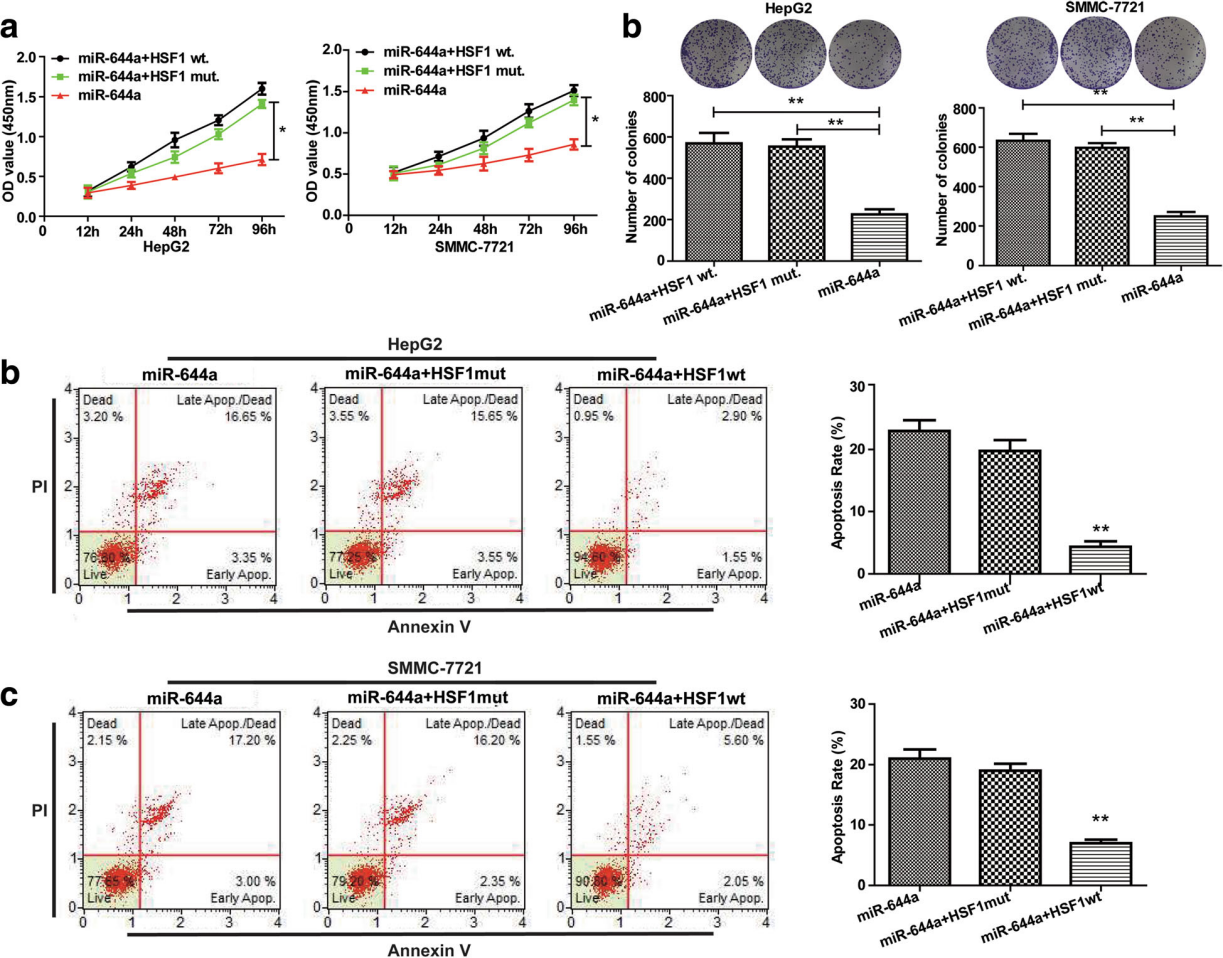
HSF1 overexpression promotes proliferation and inhibits apoptosis of HCC cells. (**a**) CCK8 assay shows proliferation of HepG2 and SMMC-7721 cells co-transfected with miR-644a mimic and wild-type or mutant HSF1 (wild type or mutant 3’UTR). (**b**) Colony formation assay shows the total number of colonies in HepG2 and SMMC-7721 cells co-transfected with miR-644a mimic and wild-type or mutant HSF1. (**c**) Flow cytometry analysis of percent apoptosis (AnnexinV^+^ PI^+^) in HepG2 and SMMC-7721 cells co-transfected with miR-644a mimic and wild-type or mutant HSF1. Note: * denotes *P* < 0.05 and ** denotes *P* < 0.01 compared to controls

Next, we analysed the proliferation, colony formation and apoptosis of HCC cells transfected with HSF1-siRNA. We observed that HSF1 silencing decreased proliferation and colony formation (Fig. 5A–B), but increased cellular apoptosis (Fig. 5C). Since miR-644a negatively regulates HSF1 expression, we postulated that up-regulation of HSF1 would increase proliferation and survival of HCC cells. Therefore, we over-expressed HCC cells with miR-644a mimic and wild type HSF1 or mutant HSF1. We observed that over-expression of wild type HSF1 increased HCC cell proliferation and colony formation (Fig. 6A–B) and decreased apoptosis (Fig. 6C). These data suggest that HSF1 up-regulation reverses the inhibitory effects of miR-644a in HCC cells.

### The miR-644a/HSF1 axis modulates expression of BH3 only apoptotic proteins

**Fig. 7:**
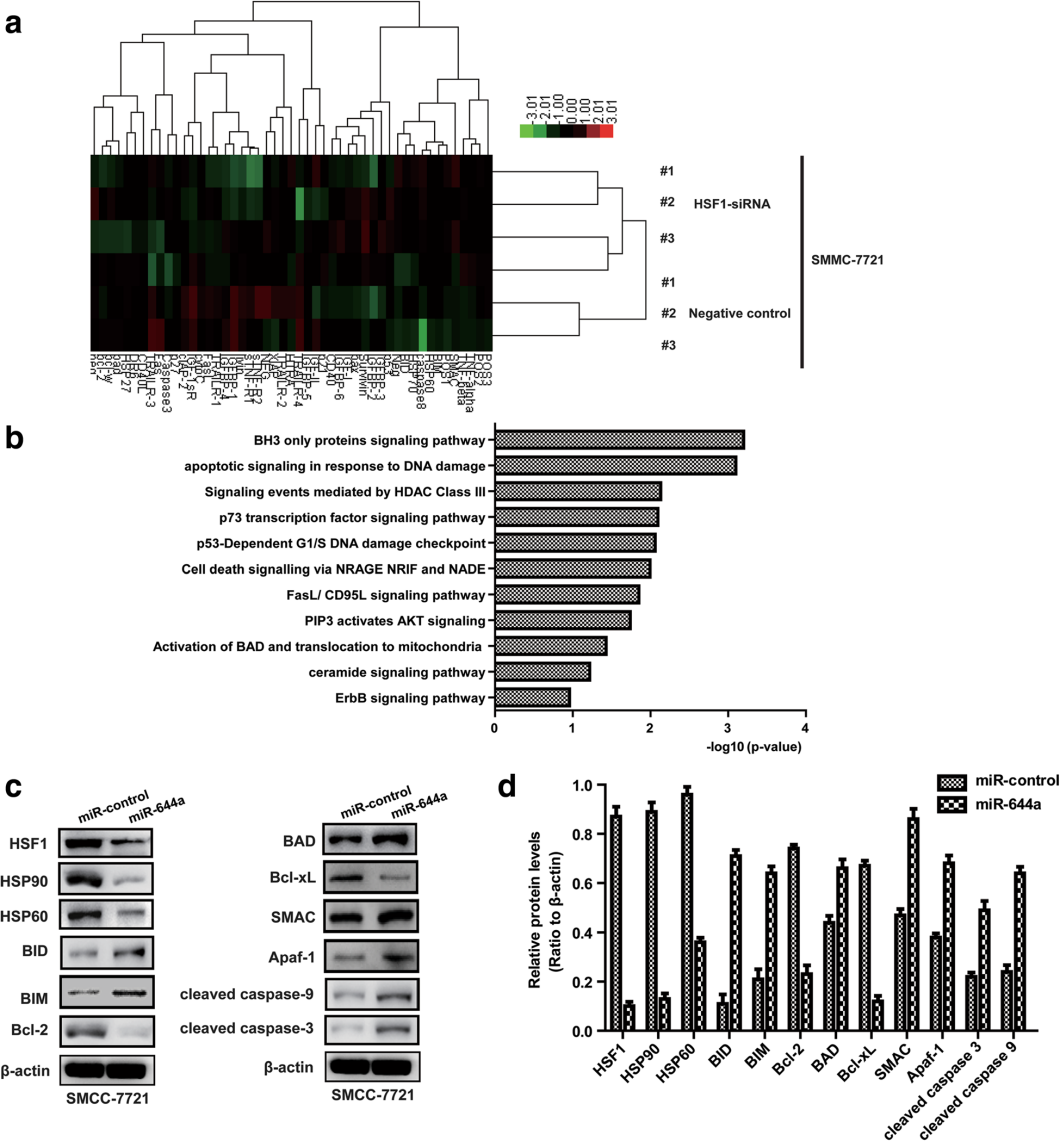
MiR-644a/HSF1 axis regulates HCC cell apoptosis via the BH3-only protein signalling pathway. (**a**) Protein chip clustering analysis shows differential protein expression in control and HSF1 siRNA transfected SMMC-7721 cells. (**b**) Protein chip enrichment analysis shows signalling pathways differentially regulated by silencing HSF1 in SMMC-7721 cells. (**c**–**d**) Representative western blots show expression of apoptosis-related proteins in SMMC-7721 cells transfected with miR-644a mimic or control miRNA. All experiments were repeated three times

To determine the role of the miR-644a/HSF1 axis in HCC, we performed protein chip analysis of apoptosis-related proteins in control and HSF1-siRNA transfected SMMC-7721 cells. HSF1 silencing resulted in changes in the expression of apoptosis-related proteins (Fig. 7A). Furthermore, HSF1 silencing up-regulated many apoptosis-related signalling pathways such as BH3-only apoptotic proteins, DNA damage signalling and HDAC class III regulated apoptotic proteins (Fig. 7B). The BH3-only apoptotic proteins were the most enriched apoptotic pathway (Fig. 7B). These results suggest that the miR-644a/HSF1 axis regulates apoptosis by modulating the expression of BH3-only proteins.

Next, we transfected SMMC-7721 cells with miR-644a mimics to down-regulate HSF1 expression and analysed the expression of BH3-only proteins by western blotting. We observed decreased expression of HSF1, HSP90, HSP60, Bcl-2, and Bcl-xL proteins and increased expression of BID, BAD, BIM, SMAC, Apaf-1 and cleaved caspases-3 and -9 (Fig. 7C). We further analysed the expression of Bcl-xl and BID in 135 HCC and adjacent normal liver patient tissues. We observed higher Bcl-xL and lower BID expression in HCC tissues than in adjacent normal liver tissues (Additional file 1: Figure S1D & Additional file 1: Figure S1E). BID expression was inversely correlated with Bcl-xL levels in HCC tissues (Additional file 1: Figure S1C). In addition, we confirmed that the BID and Bcl-xL expression levels in the xenograft model were consistent with that of the cell models (Additional file 2: Figure S2A & Additional file 2: Figure S2B). These results suggest that the miR-644a/HSF1 axis regulates BH3-only proteins in the apoptotic pathway.

### MiR-644a inhibits in vivo xenograft tumour growth by down-regulating HSF1

**Fig. 8:**
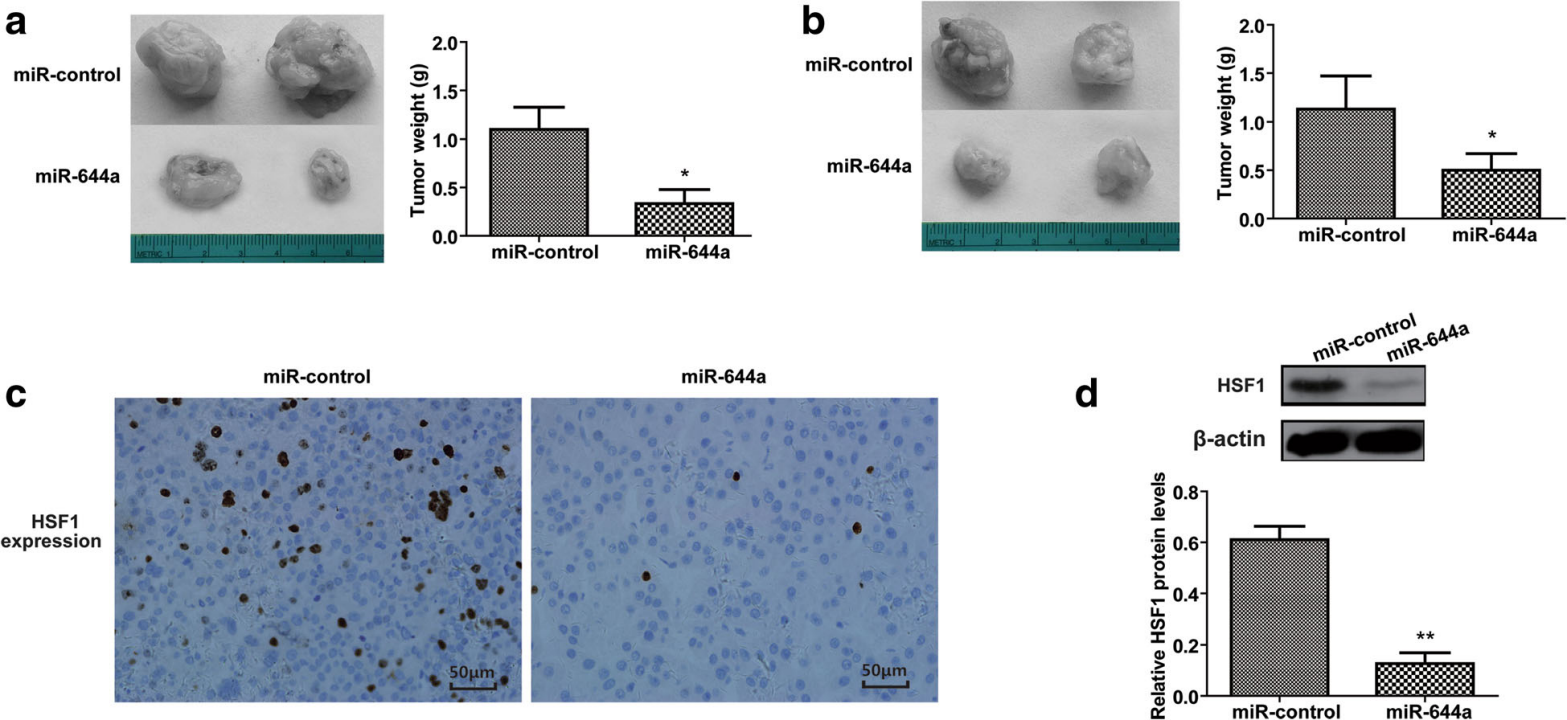
MiR-644a overexpression inhibits xenograft HCC tumour growth. (**a**) Representative images of xenograft tumours from nude mice subcutaneously injected with SMMC-7721 cells stably transfected with miR-644a mimic or control miRNA. (**b**) Xenograft tumour weights from nude mice subcutaneously injected with SMMC-7721 cells stably transfected with miR-644a mimic or control miRNA. (**c**) Representative images (400×) of IHC staining of HSF1 in xenograft tumours derived from SMMC-7721 cells stably transfected with miR-644a mimic or control miRNA. (**d**) Representative western blot shows HSF1 expression in xenograft tumours derived from SMMC-7721 cells stably transfected with miR-644a mimic or control miRNA. Note: * denotes *P* < 0.05 and ** denotes *P* < 0.01 compared to controls

**Fig. 9:**
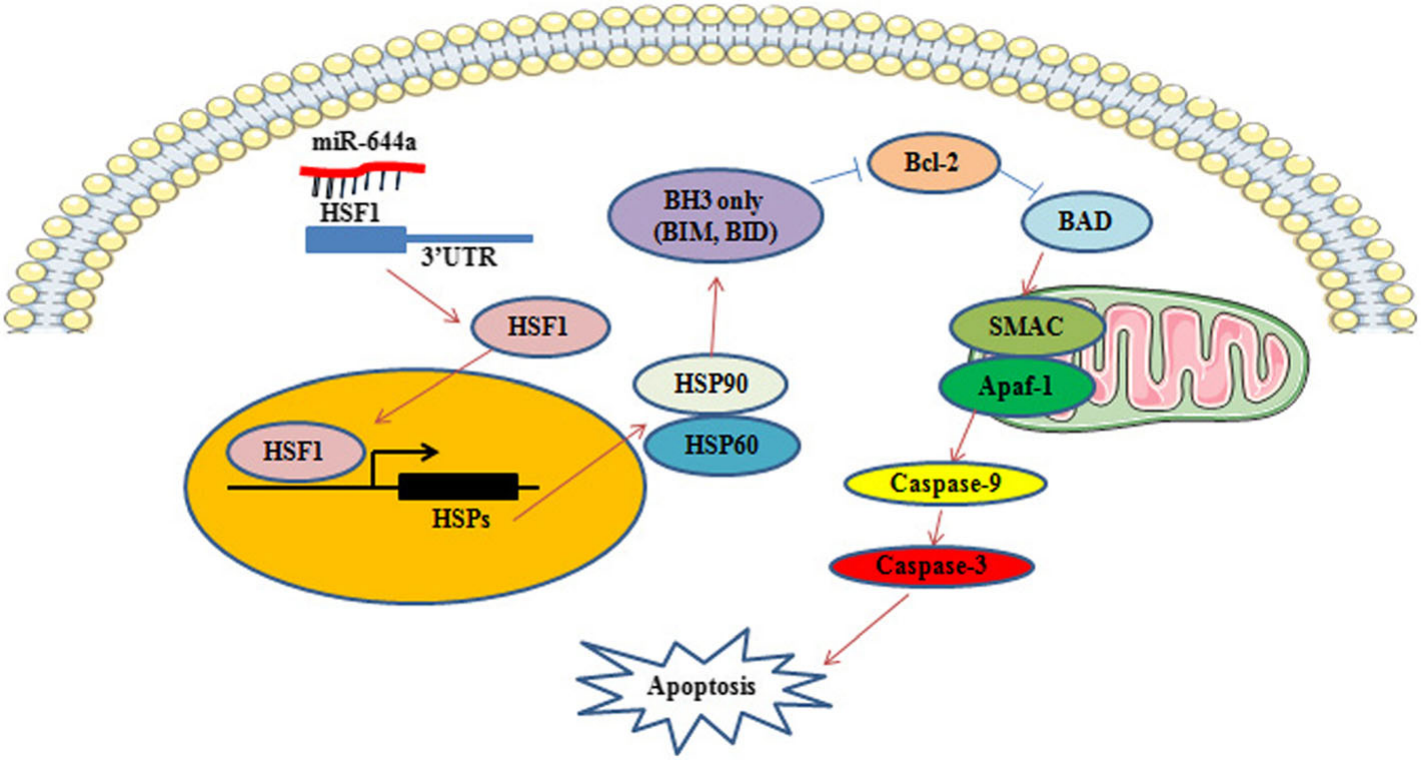
Schematic diagram of apoptosis regulation in HCC cells by miR-644a

To verify the effects of miR-644a on HCC cell proliferation in vivo, we generated stably transfected SMMC-7721 and HepG2 cell lines with miR-644a mimics and control miRNA. Then, we injected these cells into the right groin region of BALB/c nude mice and followed the tumour growth. We sacrificed the nude mice on day 35 and harvested the tumours and weighed them. We observed decreased tumour growth from HCC cells transfected with the miR-644a mimic than in the control groups (Fig. 8A–B). Subsequently, we observed low expression of HSF1 in the miR-644a mimic group tumours by IHC and western blotting (Fig. 8C–D). These results demonstrate that miR-644a inhibits HCC growth and survival by down-regulating HSF1. Thus, our results show that miR-644a inhibits HCC tumourigenesis by inhibiting HSF1 expression and promoting expression of BH3-only proteins (Fig. 9).

## Discussion

MicroRNAs are conserved small noncoding RNAs that regulate gene expression at the post-transcription or translational levels [12]. MicroRNAs also play a key role in tumourigenesis and serve as diagnostic and prognostic biomarkers as well as potential therapeutic targets [13–15]. High miRNA-644a expression inhibits the growth, progression and chemoresistance of oesophageal and breast cancers [8, 9]. However, the role of miR-644a in HCC tumourigenesis is not clear known. Our study demonstrates that miR-644a levels are low in HCC patient tissues and negatively correlated with tumour diameter and TNM stages. This suggests that miR-644a is a potential prognostic factor in HCC patients.

HSF1 is a highly conserved transcription factor that is involved in the regulation of various stress related pathways [16, 17]. Moreover, HSF1 promotes tumour cell proliferation and metabolism by cytoprotective pathways [18–20] and it can also accelerate HCC development and act as a key determinant of HCC development by regulating hepatic steatosis and metabolic syndrome [21, 22]. It had been found that ablation of HSF1 restrains the growth of c-Myc-derived mouse hepatocellular carcinoma (HCC) cell lines, where it can downregulate of c-Myc levels. Conversely, silencing of c-Myc gene in human and mouse HCC cells led to downregulation of HSF1 expression [23]. Besides, HSF1 inhibition was accompanied by downregulation of the phosphoinositide 3-kinase (PI3K)/AKT/mammalian target of rapamycin (mTOR) cascade and related metabolic pathways, so as to suppresses the growth of hepatocarcinoma cell lines [24]. MiR-34b enhances the apoptosis of acute myeloid leukaemia cells by decreasing HSF1 expression [25]. However, the role of microRNA in regulating HSF1 in HCC has not been reported. Our results demonstrate that miR-644a binds to the 3’-UTR of HSF1 in HCC cells and suppresses its expression. Subsequent induction of the expression of BH3-only proteins promotes apoptosis in HCC cells.

BH3-only proteins are a family of proteins that contain Bcl-2 homology domain 3 (BH3). They are mainly involved in cellular apoptosis and include members such as BIM, BID and NOXA [26, 27]. Excessive endoplasmic reticulum stress results in increased expression of BH3-only proteins, thereby inducing cellular apoptosis [28, 29]. BH3-only proteins down-regulate BAG3 levels and promote apoptosis of primary glioma cells [30]. Our study shows that BH3-only apoptotic pathway proteins were up-regulated by silencing HSF1 in HCC cells. Increased expression of BH3-only proteins was correlated with miR-644a over-expression. Therefore, we concluded that miR-644a promotes the apoptosis of HCC cells by down-regulating HSF1.

In conclusion, we demonstrate that miR-644a down-regulates HSF1 levels, which induces BH3-only apoptotic proteins that promote HCC cell apoptosis. Therefore, our study indicates that miR-644a is a potential prognostic biomarker and therapeutic target in HCC.

## Materials and methods

### HCC patient samples

We obtained 135 HCC patient tissue specimens and their corresponding clinical data from the Department of Hepatobiliary and Pancreatic Surgery and Department of Pathology, Affiliated Hospital of Guilin Medical University, China from 2010 to 2016. The clinical data were reviewed and approved by the Ethics Committee of Guilin Medical University. We obtained written informed consent from all of the patients in accordance with the Declaration of Helsinki. The clinicopathological data included gender, age, tumour diameter, tumour differentiation, and clinical TNM staging of the HCC patients; all patients were classified and graded based on the pTNM classification advocated by the International Union against Cancer and were followed up until June 2017.

### In situ hybridization (ISH)

The in situ hybridization assay to analyse miR-644a expression in HCC tissue samples was performed according to protocols described previously [31]. The miR-644a antisense oligonucleotide probes were obtained from Exiqon, Inc. (Woburn, MA, USA).

### Immunohistochemistry

The 135 pairs of resected HCC and para-cancerous tissues were fixed overnight with 4% paraformaldehyde, embedded in paraffin and cut into 4 mm thick sections. Immunohistochemical staining was performed with an Envision IHC kit (Maxin Biotechnologies Inc., Fuzhou, Fujian, China). Briefly, the tissue sections were dewaxed and hydrated, followed by antigen retrieval with citrate buffer for 15 min at 100 °C in a microwave oven. The sections were incubated with the primary antibody against HSF1 (Santa Cruz Biotechnology, Inc., USA) at room temperature for 1 h at a dilution of 1:200 and were visualized using the UltraVision Quanto Detection System HRP DAB kit (Thermo Scientific) according to the manufacturer’s protocols. The stained sections were counterstained with haematoxylin and then developed according to the manufacturer’s instructions and scored using an Olympus X71 microscope. Based on the staining intensity, samples were divided into the following grades: 0: < 10% positive staining HCC cells; 1+: 11–25% positive staining HCC cells; 2+: 26–50% positive staining HCC cells; 3+: > 50% positive staining HCC cells. IHC and scoring analysis were performed independently by two investigators.

### Western blotting

Total protein lysates were prepared from HCC cells and tissues in lysis buffer (50 mM Tris–HCl, 137 mM NaCl, 10% glycerol, 100 mM sodium orthovanadate, 1 mM phenylmethylsulfonyl fluoride (PMSF), 10 mg/ml aprotinin, 10 mg/ml leupeptin, 1% Nonidet P-40, and 5 mM protease inhibitor cocktail; pH 7.4), and the protein concentration was measured by a BCA (bicinchoninic acid) protein assay (Beyotime, Inc., Shanghai, China). Equal amounts of protein lysates were separated on 10% SDS-PAGE at 100 mV for 2 h. Then, the separated proteins were transferred onto PVDF membranes at 80 mV for 1 h. The blots were first blocked with 5% nonfat milk, followed by incubation with primary antibodies overnight at 4 °C. The blots were then incubated with HRP-conjugated secondary antibody at room temperature for 1 h. Then, the blots were developed by an ECL chemiluminescence method and the specific protein bands were quantified with ImageJ software. β-Actin was used as an internal control. Antibodies against HSF1, caspase 3, caspase 9, BAD, Bcl-2, Bcl-xL, BIM and BID were purchased from Santa Cruz (Dallas, Texas, USA). Antibodies against β-actin, HSP90, HSP60, SMAC and Apaf-1 were purchased from Origene (Rockville, MD, USA).

### Quantitative reverse transcription polymerase chain reaction (qRT-PCR)

Total RNA was extracted from HCC cells and tissues with TRIzol (Thermofisher Inc., Grand Island, NY, USA) and an RNAiso™ Plus kit (Takara, Japan) according to the manufacturer’s instructions. The RNA concentration was determined in a Beckman spectrophotometer (Beckman Coulter, USA). Reverse transcription was performed with the Fast Quant first strand cDNA synthesis Kit (TIANGEN, China). Then, we performed real-time PCR with Fast Start Universal SYBR Green Master Mix (Roche Diagnostics GmbH Mannheim, Germany) in an Applied Biosystems real-time PCR machine (Thermo Fisher Scientific, USA).

### Cell culture and transfections

We obtained HCC cells (HepG2, SMMC-7721, sk-Hep1, MHCC-97 L, and QGY-7701) and normal liver cells (L-O2) from the Chinese Academy of Sciences cell bank (Shanghai, China). HepG2, sk-Hep1 and MHCC-97 L were grown in Dulbecco’s modified Eagle medium (Thermo Fisher Scientific, South America), and SMMC-7721, QGY-7701 and L-O2 cells were grown in RPMI-1640 medium (Thermo Fisher Scientific) supplemented with 10% foetal bovine serum (Thermo Fisher Scientific) at 37 °C and 5% CO_2_. The transfection of miR-644a mimic and HSF1-siRNA were prepared and used according to a previously described protocol [32].

### CCK-8 cell proliferation assay

HCC cells were seeded in 96-well plates (3 × 10^3^ cells in 100 μl per well). At different time points, 10 μl of cell counting kit-8 (CCK-8) solution (Dojindo, Shanghai, China) was added and incubated for 1–3 h. Absorbance was read at 450 nm in a microplate reader (Spectramax plus384, Molecular Devices, USA).

### Flow cytometry analysis of apoptosis

After various treatments, HCC cells were washed twice with 1× PBS and centrifuged. Then, 100 μl of FITC-conjugated Annexin-V and PI (Sigma, USA) was added and the cells were incubated for 20 min at room temperature in the dark. Then, the cells were subjected to flow cytometric analysis (Merck Millipore, Germany). The percentage of apoptotic cells (Annexin V+ PI+) was determined by using FlowJo software 7.6 (Treestar, USA).

### Colony formation assays

We seeded 600 HCC cells per well in a six-well plate for approximately two weeks in 10% DMEM or RPMI medium supplemented with 10% foetal bovine serum. Then, the colonies were fixed with 4% paraformaldehyde for 20 min and stained with 1% crystal violet (G1062, Solarbio, Japan) overnight. After washing the cells three times, the total number of colonies (> 10 cells per colony) per well was determined for all conditions. Images were also captured for documentation.

### Dual luciferase reporter assay

We identified the miR-644a target site in the HSF1–3’UTR with TargetScan 6.2 (http://www.targetscan.org/vert_71/). We then designed primers to generate the mutant and wild type HSF1 based on the HSF1 mRNA sequence in NCBI GenBank. We PCR amplified the miR-644a target sequence in HSF1 from the total RNA from the HCC cells. The mutant version of the HSF1 3’UTR was generated by overlapping PCR with mutagenic primers. We cloned both wild-type and mutant HSF1–3’UTR sequences into the pMIR-REPORT-basic vector (Applied Biosystems, USA) and confirmed the products by DNA sequencing. For the luciferase reporter assay, we seeded 3 × 10^3^ cells in 24-well plates and co-transfected 100 ng of the luciferase reporter vector with 20 nM miR-644a mimic or miRNA negative control into SMMC-7721 cells. The cells were lysed after 48 h, and the dual luciferase activities were determined using the luminescence reporter gene assay system (PerkinElmer, Norwalk, CT, USA) according to the manufacturer’s instructions.

### Generation of stable miR-644a over-expressing HCC cell lines

We purchased lentivirus pEZX-MR04 plasmid clones with miR-644a mimics and negative control miRNA from GenePharma (Shanghai, China) and transfected them into HEK293T cells with EndoFectin Lenti transfection reagent (GeneCopoeia, Rockville, USA). The cells were centrifuged after 48 h at 10000 rpm for 10 min, and the lentiviral particles expressing miR-644a or negative control miRNAs in the supernatant were concentrated by ultrafiltration. Then, SMMC-7221 cells were infected with lentiviruses expressing miR-644a or negative control miRNAs in the presence of 2 μg/ml puromycin and cultured for 2 weeks to obtain stably transfected cells. The expression of miR-644a in the stably transfected control and miR-644a overexpressing SMMC-7221 cells was determined by qRT-PCR.

### Xenograft nude mice model of in vivo tumourigenesis

We purchased forty 5–6-week-old male BALB/c nude mice from the Animal Experimental Center of Guilin Medical University. All animal experiments were approved by the Animal Care and Use Committee of Guilin Medical University. We randomly divided the nude mice into two groups and injected them subcutaneously into the right inguinal region with 200 μl (2 × 10^7^) of SMMC-7221 cells that were stably transfected with either empty plasmid (control) or miR-644a mimic (experimental). Tumour growth was recorded at 7, 14, 21, 28 and 35 days. Then, the mice were sacrificed by cervical dislocation on day 35; the tumours were harvested by resection and weighed.

### Protein chip analysis

We incubated the protein samples isolated from miR-644a overexpressing and control SMMC-7221 cells with the antibody chip AAH-APO-G1–8 (RayBiotech, Norcross, GA, USA). Then, the unbound proteins were removed by washing and the bound proteins were analysed by fluorescence scanning. We determined the differential expression of the proteins and performed clustering analysis with Cluster v3.0 software (Stanford University, USA). Then, we annotated the differentially expressed proteins and determined the molecular networks by gene ontology and KEGG enrichment analysis.

### Statistical analysis

The statistical analysis was performed with GraphPad Prism 5 (GraphPad Software, Inc., San Diego, CA, USA) and SPSS v.18.0 (SPSS Inc., Chicago, IL, USA). The relationship between miR-644a expression and clinicopathological data was evaluated by the chi-square test (χ^2^). Quantitative data was analysed by the paired t test. Kaplan-Meier analysis was performed to estimate the survival rate and prognosis of patients with HCC expressing high or low miR-644a and HSF1 levels. The logarithmic rank test was used to compare the survival curves between different groups. All experiments were repeated three times, and the data are expressed as the mean ± SD. *P* < 0.05 was considered statistically significant.

## Additional files

Additional file 1: **Figure S1.** (**A**–**B**) ROC curve analysis of miR-644a and HSF1 expression in HCC tissues. (**C**) Correlation between BID and Bcl-xL expression in HCC tissues by immunohistochemistry. (**D**–**E**) Representative images (× 200) show immunohistochemical analysis of Bcl-xL and BID expression in HCC and adjacent peri-cancerous tissues. Note: *** denotes *P* < 0.001 when compared to adjacent tissues. (PDF 6242 kb)

Additional file 2: **Figure S2.** (**A**–**B**) Representative images (400×) of IHC staining of BID and BcL-xL in xenograft tumours derived from SMMC-7721 cells stably transfected with miR-644a mimic or control miRNA. Note: *** denotes *P* < 0.05 when compared to adjacent tissues. (TIF 3954 kb)
