# Correction: MicroRNA-644a promotes apoptosis of hepatocellular carcinoma cells by downregulating the expression of heat shock factor 1

**DOI:** 10.1186/s12964-022-00975-4

**Published:** 2022-09-22

**Authors:** Wenjin Liang, Yong Liao, Zeming Li, Yan Wang, Siqi Zheng, Xiaochen Xu, Fulin Ran, Bo Tang, Zhenran Wang

**Affiliations:** Department of Gastrointestinal Surgery and Hepatobiliary Surgery, Guilin Medical University, Affiliated Hospital, Guilin, 541001 Guangxi People’s Republic of China

## Correction to: Cell Communication and Signaling (2018) 16:30 https://doi.org/10.1186/s12964-018-0244

Following publication of the original article [1], the authors reported errors that were inadvertently introduced in Figs. 5 and 6. Specifically, the icons of b and c in Figs. 5 and 6, as well as the flow cytometry analysis of HepG2 and SMMC-7721 cells transfected with HSF1-siRNA in Fig. 5 and the SMMC-7721 cells transfected with miR-644a+ HSF1 mut. In Fig. 6 are incorrect in the original publication. The authors sincerely apologize for the error and confirm that this correction does not change the conclusion of the article. The corrected images has been replaced as follows:

**Fig. 5:**
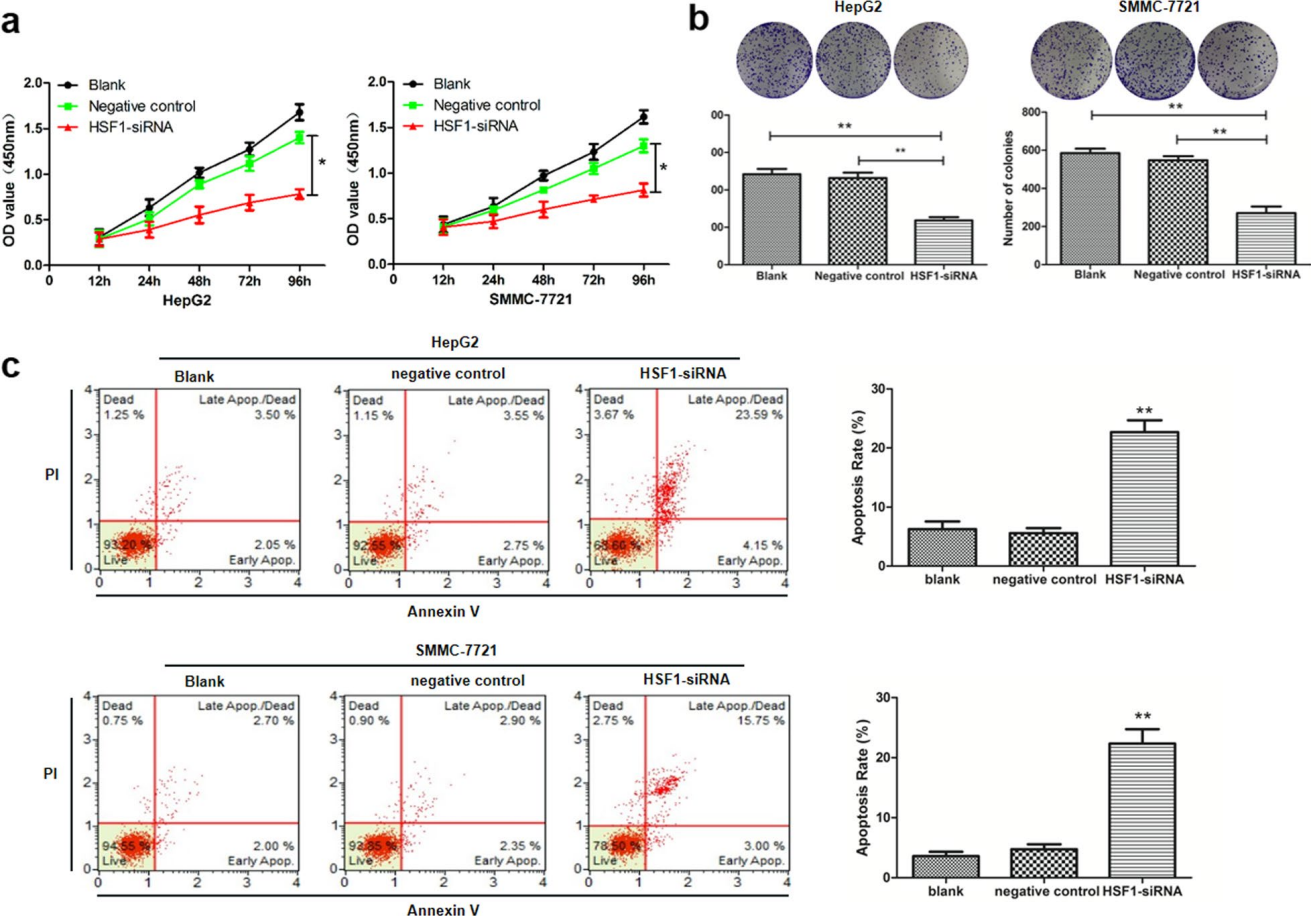
HSF1 silencing decreases proliferation and increases apoptosis of HCC cells. **a** CCK8 assay shows proliferation of HepG2 and SMMC-7721 cells transfected with control or HSF1-siRNA. **b** Colony formation assay shows the total number of colonies formed by HepG2 and SMMC-7721 cells transfected with control or HSF1-siRNA. **c** Flow cytometry analysis shows apoptosis of HepG2 and SMMC-7721 cells transfected with control or HSF1-siRNA. *Note*: * denotes *P* < 0.05 and ** denotes *P* < 0.01 compared to controls

**Fig. 6:**
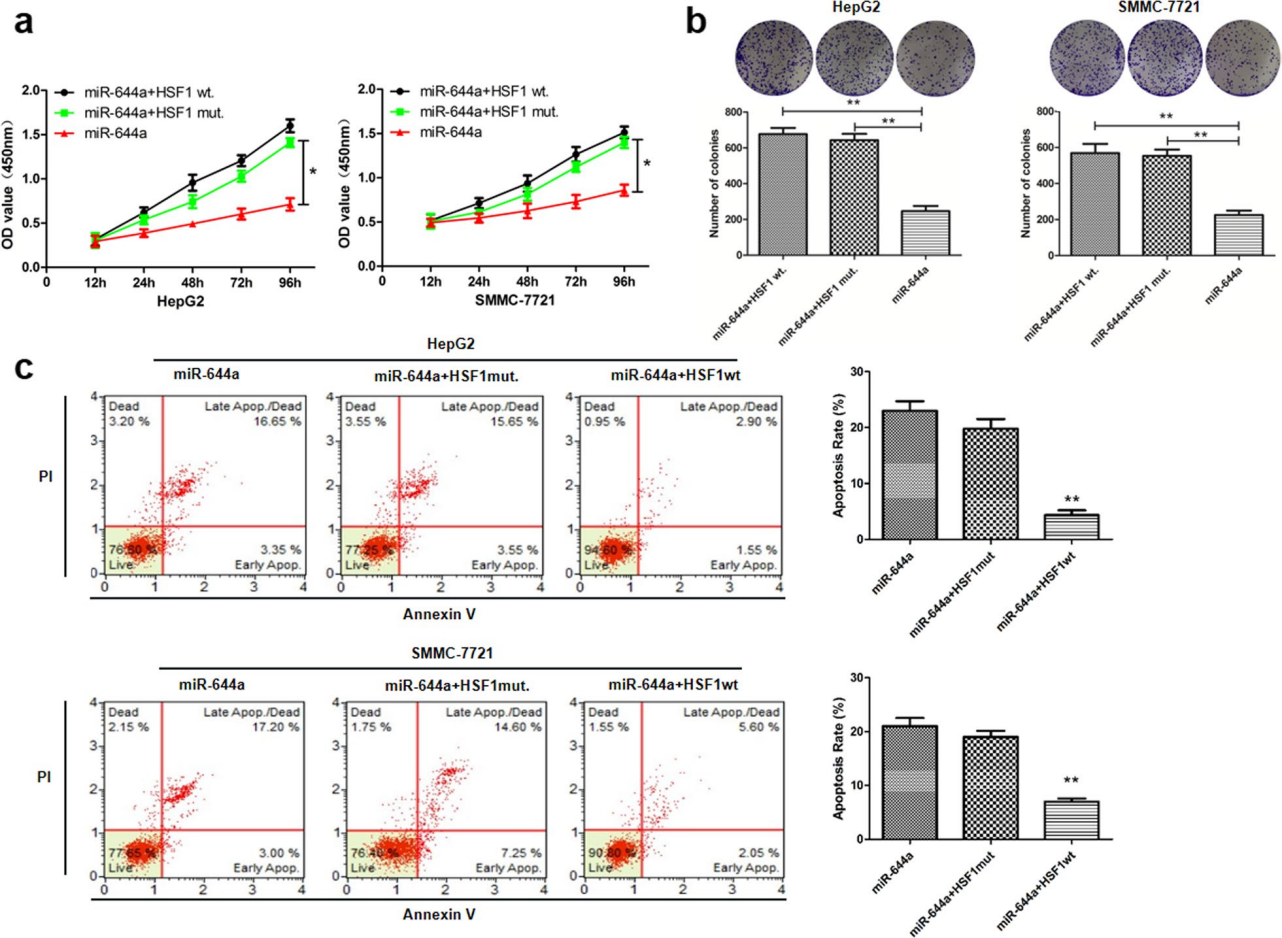
HSF1 overexpression promotes proliferation and inhibits apoptosis of HCC cells. **a** CCK8 assay shows proliferation of HepG2 and SMMC-7721 cells co-transfected with miR-644a mimic and wild-type or mutant HSF1 (wild type or mutant 3’UTR). **b** Colony formation assay shows the total number of colonies in HepG2 and SMMC-7721 cells co-transfected with miR-644a mimic and wild-type or mutant HSF1. **c** Flow cytometry analysis of percent apoptosis (AnnexinV^+^ PI^+^) in HepG2 and SMMC-7721 cells co-transfected with miR-644a mimic and wild-type or mutant HSF1. *Note*: * denotes *P* < 0.05 and ** denotes *P* < 0.01 compared to controls

## References

[1] Liang W, Liao Y, Li Z (2018). MicroRNA-644a promotes apoptosis of hepatocellular carcinoma cells by downregulating the expression of heat shock factor 1. Cell Commun Signal.

