# Supplementary figures and images for: MicroRNA-644a promotes apoptosis of hepatocellular carcinoma cells by downregulating the expression of heat shock factor 1
Source: Cell Commun Signal. 2018 Jun 14;16:30. doi: 10.1186/s12964-018-0244-z (PMC6001142; doi:10.1186/s12964-018-0244-z)

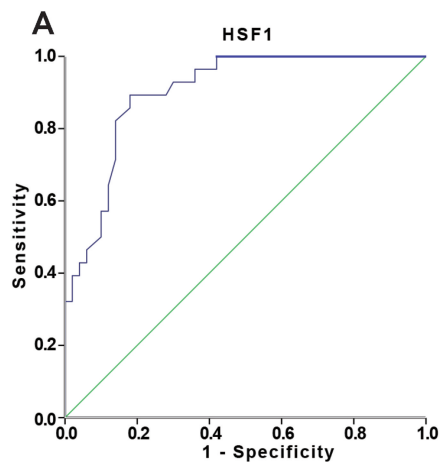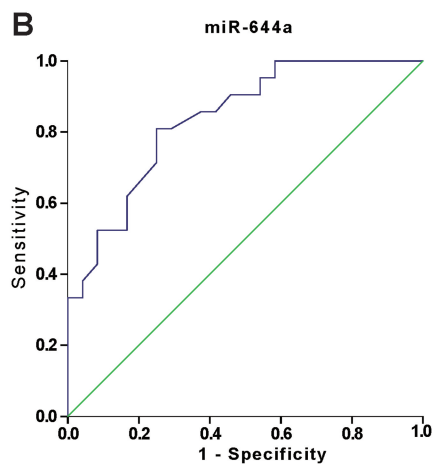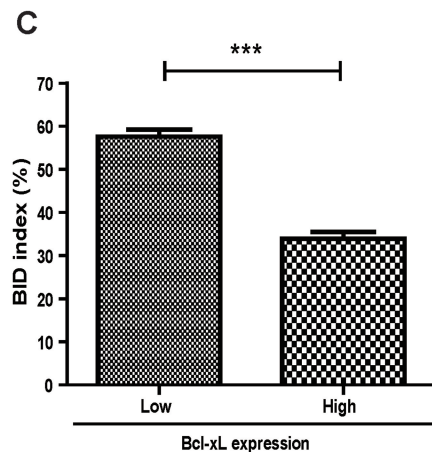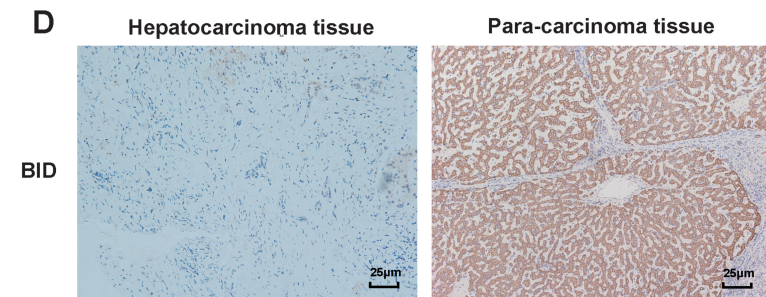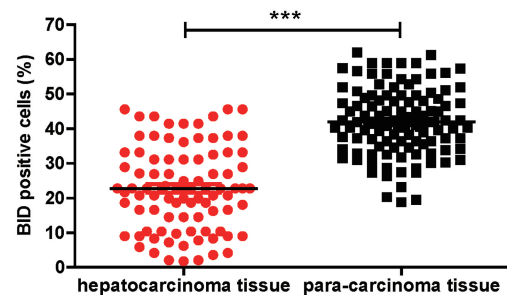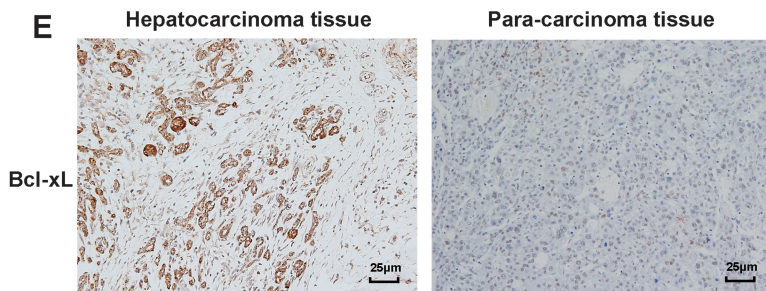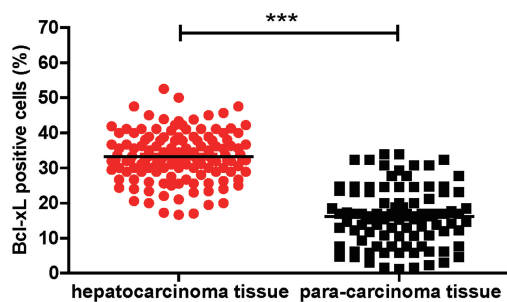

Supplement: Supplementary file 1 — Figure S1. (A–B) ROC curve analysis of miR-644a and HSF1 expression in HCC tissues. (C) Correlation between BID and Bcl-xL expression in HCC tissues by immunohistochemistry. (D–E) Representative images (× 200) show immunohistochemical analysis of Bcl-xL and BID expression in HCC and adjacent peri-cancerous tissues. Note: *** denotes P < 0.001 when compared to adjacent tissues. (PDF 6242 kb) [file 12964_2018_244_MOESM1_ESM.pdf]

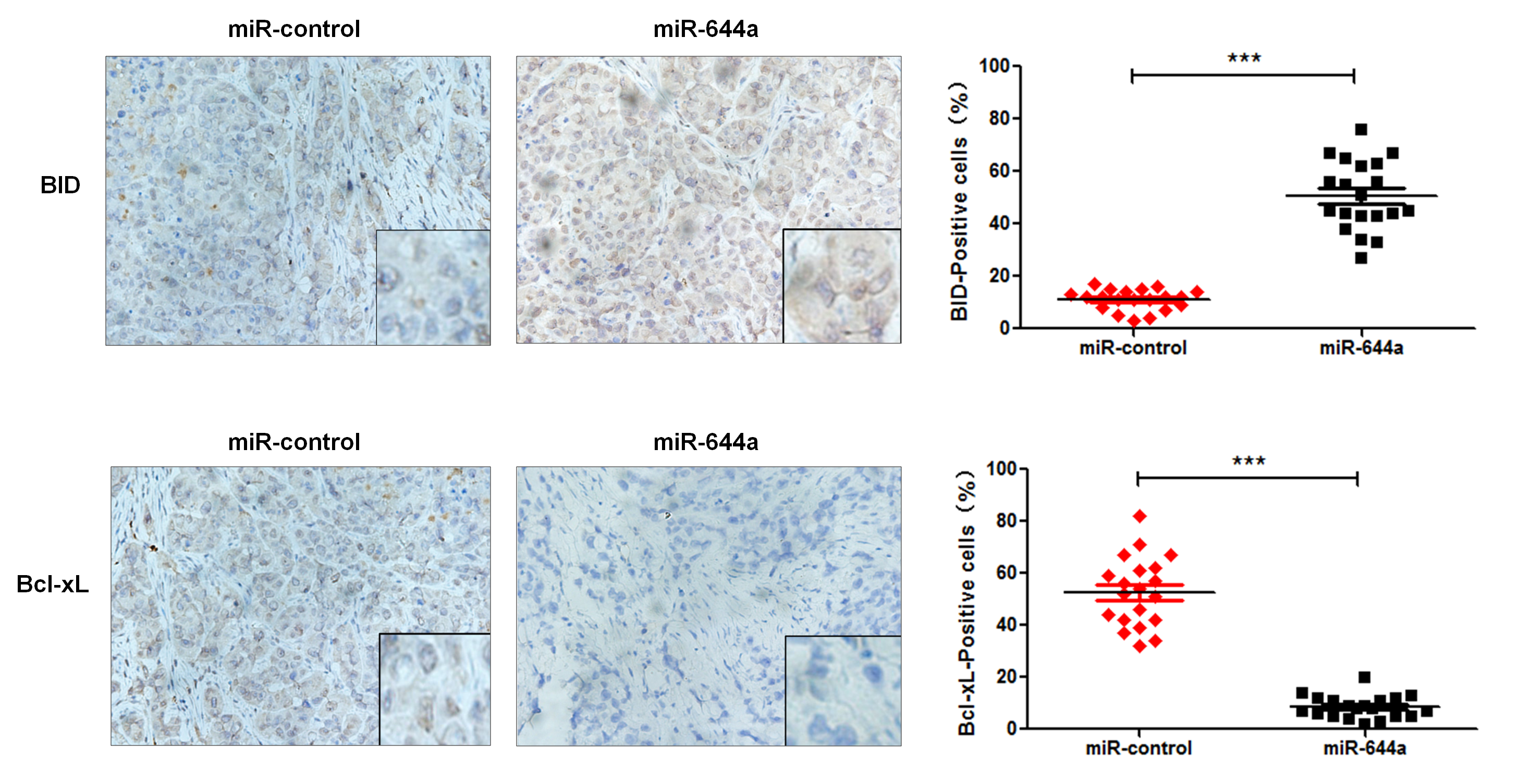

Supplement: Supplementary file 2 — Figure S2. (A–B) Representative images (400×) of IHC staining of BID and BcL-xL in xenograft tumours derived from SMMC-7721 cells stably transfected with miR-644a mimic or control miRNA. Note: *** denotes P < 0.05 when compared to adjacent tissues. (TIF 3954 kb) [file 12964_2018_244_MOESM2_ESM.tif]
